# Gradient based spinal cord axogenesis and locomotor connectome of the hatchling *Xenopus* tadpole

**DOI:** 10.1186/1471-2202-12-S1-O9

**Published:** 2011-07-18

**Authors:** Abul Kalam al Azad, Roman Borisyuk, Alan Roberts, Steve Soffe

**Affiliations:** 1 School of Computing and Mathematics, University of Plymouth, Plymouth, PL48AA, UK; 2 Institute of Mathematical Problems in Biology, Pushchino, Moscow Region, 142290, Russia; 3 School of Biological Sciences, University of Bristol, Bristol, BS81UG, UK

## 

Understanding the mechanisms underlying the self-assembly and organization of functional neuronal networks is a crucial problem confronting both experimental and theoretical neuroscience alike. Early in development, functional neuronal networks self-assemble with astonishing rapidity. It is, therefore, imperative to investigate and understand how far simple basic mechanisms can allow primary functioning neuronal circuits to develop. To address this ‘structure-function’ issue, we model anatomy and electrophysiology of young hatchling Xenopus tadpole’s spinal cord [1-3]. Our bottom-up approach to modeling of neuronal connectivity is based on developmental process of axon growth – we develop a gradient-based mathematical model for axon growth. It is known that in the developing vertebrate spinal cord, neurons arise from progenitor cells in the neural tube and thereafter the axons grow under influence of chemical morphogenes released from the dorsal roof plate (‘BMP’), ventral floor plate (‘shh’) and hindbrain regions (‘Wnt’). Distribution of these guidance molecules along the spinal cord set up a gradient field which steer the axons in appropriate locations and thus ensure formation of proper connections. We grow axons of spinal neurons and generate synaptic connections similar to biological developmental process based on the data from Professor Alan Roberts Lab at University of Bristol [4]. Using the gradient-based model we were able to grow axons for all seven types of spinal neurons which are believed to be involved in swimming and struggling behavior of tadpole. These spinal neurons include sensory neurons which are responsible for receiving stimulus from environment; interneurons which process the sensory information and pass it onto the motor which translate these signals into appropriate locomotive activities. We successfully modeled both morphologically different types of spinal neurons– firstly commissural neurons grow axons ventrally on the same side of the spinal cord at first and turn longitudinally on the other (contralateral) side of the spinal cord, whereas non-commissural neurons grow their axons on the same (ipsilateral) side of spinal cord. The model incorporates experimental data for somata distribution in the spinal cord, the outgrowth angles, axon lengths, etc. The computer modeling of the axon growth of spinal neurons has enabled both us and our Biology partners to construct and test various hypotheses particularly the roles of the different guidance cues in the axon growth. We also implemented an optimization technique to determine the model parameters for a particular type of neuron based on minimizing a cost function which comprises of the difference between the axon trajectory distribution of the model axons and the corresponding experimental data and also difference between the tortuisities of the model and experimental axons. The complete reconstruction of the spinal cord includes about 2000 neurons. In the reconstruction, both the neurons and the dendrites are randomly distributed along the spinal cord on the ‘left’ and ‘right’ side of the body according to the experimental measurements. Thus, the reconstruction of the connectivity is biologically realistic. To model spiking activity we use Hodgkin-Huxley type conductance model. Parameters of the model are chosen according to the available neurophysiological measurements. Activity dynamics of neural network comprising both the reconstruction model and conductance based spiking neurons demonstrates swimming patterns, i.e., anti-phase oscillations on opposite sides of the body and metachronal wave in longitudinal direction in a wide range of model parameters.
